# Combined Influence of Subjective Cognitive Complaints and Neuropsychiatric Symptoms on Cognitive Trajectories in Older Adults

**DOI:** 10.3390/brainsci16070693

**Published:** 2026-06-30

**Authors:** Cindy Zhang, Ashleigh S. Vella, Russell J. Chander, Henry Brodaty, Perminder S. Sachdev, Katya Numbers

**Affiliations:** 1 Centre for Healthy Brain Ageing (CHeBA), Discipline of Psychiatry & Mental Health, School of Clinical Medicine, Faculty of Medicine & Health, University of New South Wales, Sydney, NSW 2033, Australia; 2 The Neuropsychiatric Institute, The Prince of Wales Hospital, Randwick, NSW 2031, Australia

**Keywords:** dementia, neuropsychiatric symptoms, cognition, dementia, subjective cognitive complaint

## Abstract

**Highlights:**

**What are the main findings?**

- Individuals with neuropsychiatric symptoms had significant associations with domain-specific cognitive outcomes, including visuospatial ability and executive function, and global cognition.
- Having both subjective cognitive complaints and neuropsychiatric symptoms was associated with elevated risk of incident dementia, more so than having either symptom.

**What are the implications of the main findings?**

- Clinical screening of neuropsychiatric symptoms in non-demented individuals may allow early identification of subtle cognitive vulnerabilities and poor future global cognitive performance.
- The co-occurrence of SCC and NPS may provide useful risk information in older adults, although predictive accuracy and clinical utility require further validation.

**Abstract:**

**Objective:** Subjective cognitive complaints (SCC) and neuropsychiatric symptoms (NPS) are both recognized as early markers of dementia, but their combined predictive value is not well understood. This study investigated whether SCC and NPS, individually and jointly, predicted six-year cognitive decline and 12-year incident dementia in community-dwelling older adults. **Methods:** Participants were 468 dementia-free individuals aged 70–90 from the Sydney Memory and Ageing Study, followed for up to 12 years. Cognitive decline was assessed biennially via neuropsychological tests; dementia diagnoses were made by expert consensus. SCC were captured using two self-reported and one informant-reported item. NPS were assessed using the informant-rated Neuropsychiatric Inventory. SCC and NPS were each operationalised as ordinal scores ranging from 0 to 3, with higher scores reflecting more SCC items or more NPS clusters, respectively, and participants were categorized into SCC−/NPS−, SCC+/NPS−, SCC−/NPS+, SCC+/NPS+. Linear regression models assessed associations with six-year cognitive decline across domains and global cognition, and Cox proportional hazards models assessed incident dementia risk over 12 years. **Results:** SCC sum scores predicted dementia risk, whereas NPS sum scores were not significantly associated with dementia risk. In the combined SCC/NPS group analyses, SCC+/NPS+ participants had a 66% higher hazard of dementia (HR = 1.66, *p* = 0.041). For cognitive decline, the SCC−/NPS+ group showed significantly greater decline in executive function, visuospatial ability, and global cognition compared with SCC−/NPS−. Neither SCC+/NPS− nor SCC+/NPS+ predicted domain-specific decline. **Discussion:** SCC and NPS showed different patterns of association with later outcomes, with the combined SCC/NPS profile identifying a subgroup at elevated dementia risk. These findings support closer monitoring of older adults who present with co-occurring SCC and NPS, as this profile may help improve early identification of individuals at risk of adverse cognitive outcomes.

## 1. Introduction

Dementia develops over decades before a diagnosis can be made. In the years preceding measurable objective decline, cognition may appear intact despite progressive underlying pathology, creating a critical but elusive window for early intervention. Identifying reliable, non-invasive markers of risk during this preclinical phase remains one of the central challenges in dementia research [1]. In community-based cohorts, Alzheimer’s disease (AD) and vascular dementia (VaD) account for most cases, yet both share this extended prodromal trajectory, underscoring the need for markers that can be applied at scale, before clinical thresholds are met.

### 1.1. Subjective Cognitive Decline

Cognitive impairment exists on a continuum of severity, with the stage preceding dementia termed mild cognitive impairment (MCI) [1,2]. Researchers are increasingly focusing on earlier, pre-MCI stages, where pathology exists in the absence of impaired cognitive performance [1,3]. Subjective cognitive decline (SCD) has gained traction as a pre-MCI stage of dementia over the last decade [1,3]. SCD is defined by consistent self- or informant-reported subjective cognitive complaints (SCC) despite normal performance on standardized cognitive tests [1,3,4]. In this manuscript, SCD is used to refer to the broader clinical and research construct, while SCC refers to the specific subjective cognitive complaint items measured in the present study. Recent biomarker studies suggest that SCD may, in some individuals, reflect early Alzheimer’s disease pathology, with associations reported between SCD and amyloid burden, regional tau deposition, and plasma markers of amyloid, tau, neuroaxonal injury, and glial activation [5,6].

Similarly, meta-analytic and longitudinal evidence consistently show that SCD is associated with increased risk of MCI and dementia [7,8], but this risk signal is not specific to neurodegeneration. That is, in cognitively normal adults, SCD often covaries with mood, personality traits, and psychological distress rather than objective cognitive performance [7,9,10].

### 1.2. Neuropsychiatric Symptoms

NPS are behavioural and psychological disturbances that can occur in tandem across a range of psychiatric and neurological conditions, as well as in cognitively healthy older adults [11]. NPS are particularly common and often associated with greater cognitive and functional impairment in individuals with dementia. While NPS are well-established contributors to decline in overall cognitive performance, domain-specific patterns vary across studies [12,13]. These between-study differences likely reflect variation in disease stage of the cohorts and how NPS and cognitive domains were measured, underscoring an area for further research. Notably, several large longitudinal studies indicate that NPS increases the risk of progressing to dementia from MCI [14,15]. Although certain symptoms, such as anxiety and apathy, were linked to a higher risk of developing dementia, these varied depending on the study’s follow-up periods and operationalisation of NPS.

More recently, NPS have also been conceptualised within the framework of mild behavioural impairment (MBI), which refers to later-life, sustained behavioural and psychological symptoms that may represent an early manifestation of neurodegenerative disease [11]. Overall, evidence for associations between NPS and cognitive performance and decline is consistent, but domain-specific patterns vary across studies [12,13,16,17]. For example, one study reported that anxiety was related to a decline in memory, attention, and executive function, while another reported that depression, anxiety, and apathy were associated with worsening episodic memory [14,15].

### 1.3. Combined Utility of SCD and NPS

To our knowledge, only one study has examined whether the co-occurrence of cognitive complaints and behavioural symptoms provides additive predictive power to confer dementia risk [18]. In their study, participants were assigned to one of four groups based on the absence or presence of SCD and MBI: SCD−/MBI−, SCD+/MBI−, SCD−/MBI+, and SCD+/MBI+. The SCD+/MBI+ group showed the highest risk of progression to dementia, suggesting an additive predictive effect. However, their study had a relatively short follow-up period (three years) and required longitudinal data to determine MBI. While they considered the risk of incident MCI and dementia, they did not assess changes in cognitive domains.

### 1.4. Present Study

The present study extends the work of Ismail et al. [18] by examining whether the co-occurrence of SCC and NPS provides additive predictive utility for cognitive decline and dementia risk. Most prior work has focused on short- to medium-term cognitive trajectories, and we extend this by evaluating both (i) six-year cognitive domain decline and (ii) 12-year dementia incidence. To achieve this, we analysed SCC and NPS using simplified ordinal scores derived from brief binary SCC items and NPI symptom clusters, rather than applying formal SCD or MBI diagnostic criteria. This approach reflects the type of information commonly available in large epidemiological cohorts and may support pragmatic identification of risk profiles across studies. We hypothesised that SCC and NPS scores were associated with increased dementia risk. Additionally, we expected that having both SCC and NPS would predict (i) steeper cognitive decline and (ii) greater dementia risk than either SCC or NPS alone.

## 2. Methods

### 2.1. Participants

Participant data were obtained from the Sydney Memory and Ageing Study (MAS). Between 2005 and 2007, MAS randomly recruited individuals aged 70–90 using the electoral roll from two electorates in Sydney’s South-Eastern Suburbs. Exclusion criteria included insufficient English proficiency, a baseline dementia diagnosis, or a Mini-Mental State Examination (MMSE) score below 24, or other specified medical or psychiatric conditions. After exclusions, the final MAS baseline sample comprised 1037 individuals (see [19] for further details on recruitment).

Participants were assessed biennially (called “Waves”) by trained research assistants. At each Wave, participants completed questionnaires regarding demographics, medical history, mood, and lifestyle information. Additionally, they underwent in-person assessments, which included comprehensive neuropsychological testing, a brief medical exam, and blood samples (Waves 1, 3, and 4). Furthermore, participants nominated an informant with whom they shared at least one hour of weekly contact and who had adequate knowledge of the participant’s function and any relevant changes. Informants completed questionnaires about the participant’s health, mood, cognitive changes, and functional ability over the telephone. Dementia was diagnosed via clinical consensus based on the Diagnostic and Statistical Manual of Mental Disorders, Fourth Edition (DSM-IV), which requires impairment in the domain of memory and in one other cognitive domain, severe enough to impact daily function [20].

**Figure 1:**
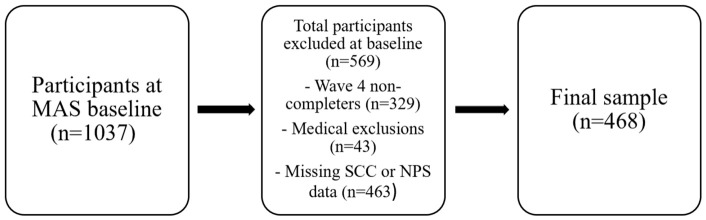
Flow chart showing the process to achieve the final sample size that was included in both the 6-year and 12-year analyses.

The present study drew data from the full MAS baseline sample of 1037 participants. To be included, participants were required to have completed cognitive assessments at Wave 4 (six-year follow-up; 2011–2013; N = 708), as this was the latest wave with detailed domain-specific cognitive data. Additional exclusion criteria were incomplete data for main predictor and outcome variables (i.e., SCC, NPS, cognitive domains, dementia), and current major medical issues at baseline (e.g., active cancer, brain tumour, etc.). After exclusions, the final sample for the present study comprised 468 older adults (see Figure 1). These same 468 participants contributed to both the six-year cognitive analysis and the twelve-year dementia outcome analysis. This extended the observation window for clinical progression beyond the cognitive characterisation period. Written informed consent was provided by all participants and informants, and the study was approved by the University of New South Wales Human Research Ethics Committee (HC: 05037, 09382, 14327, 190962) in accordance with the National Statement on Ethical Conduct in Human Research [21] and the Declaration of Helsinki. Informed written consent was obtained from all participants prior to participation.

### 2.2. Measures

#### 2.2.1. Subjective Cognitive Complaints

SCCs were assessed using three binary (yes/no) items, two completed by the participants and one by the informant. SCC item 1 asked, “Have you noticed a change in your memory in the last five years?” If participants reported yes, they would move to SCC item 2, where they were asked, “Have you been concerned about the change you’ve noticed in your memory in the last five years?” SCC item three required informants to respond to the question, “Have you noticed a change in the participant’s memory in the last five years?” For each item, a “yes” response was coded as 1, and a “no” response was coded as 0. Total SCC sum scores were calculated for each participant and ranged from 0 to 3.

#### 2.2.2. Neuropsychiatric Symptoms

The Neuropsychiatric Inventory (NPI) assessed the presence or absence of 12 NPS, including delusions, hallucinations, dysphoria, anxiety, agitation, euphoria, disinhibition, irritability, apathy, aberrant motor activity, sleep disturbances, and appetite abnormalities. For each NPS, informants first answered a binary screening question; if “yes”, they completed follow-up questions to confirm the symptom presence. A symptom was endorsed only if the follow-up responses confirmed its presence; otherwise, it was coded as absent.

Based on the exploratory factor analysis conducted by Sayegh and Knight [22], 10 of the original 12 NPI items were retained and grouped into three clusters: psychotic, affective, and hyperactivity (Table 1). The two items not included in their factor solution (elation and aberrant motor behaviour) were also excluded from our analyses. In line with their findings, these symptoms had very low prevalence (elation with 0.4% and aberrant motor behaviour with 0.6%) in our sample, further supporting their exclusion.

For each cluster, participants received a score of 1 if they endorsed at least one symptom within that cluster, and 0 if none of the symptoms were endorsed. As with SCC scores, NPS cluster scores were summed and ranged from 0 to 3.

#### 2.2.3. Group Status

In addition to SCC and NPS sum scores, participants were also categorized into four groups based on the endorsement of any SCC question or any NPS cluster. For example, for SCC, a score of 1 on any of the three items (participant report, participant concern, or informant report) was coded as SCC+. For NPS, a score of 1 in any of the three symptom clusters (psychotic, affective, or hyperactivity) was coded as NPS+. This resulted in four mutually exclusive groups: SCC−/NPS− (absence of both SCC and NPS), SCC+/NPS+ (presence of both SCC and NPS), SCC−/NPS+ (NPS present, SCC absent), or SCC+/NPS− (SCC present, NPS absent).

#### 2.2.4. Cognitive Domains

At each Wave, participants completed a comprehensive neuropsychological battery that captured six cognitive domains: attention/processing speed, language, executive function, visuospatial, and verbal memory. The battery was comprised of Digit Symbol-Coding, Trail Making Test A, Trail Making Test B, Logical Memory Story A delayed recall, Rey Auditory Verbal Learning Test, Benton Visual Retention Test recognition, Boston Naming Test, Semantic Fluency (Animals), Block Design, and Controlled Oral Word Association Test [19]. The tests and their corresponding cognitive domains are indicated in Supplementary Table S1. Scores were standardised as quasi-z-scores using the baseline mean and standard deviations of a healthy MAS subsample. The mean of participants’ quasi-z-scores in tests relevant to a specific domain formed the composite domain scores. A global cognition score was then calculated using the mean of five domain z-scores. As cognitive domain data until W4 (6 years after baseline) were available at the time of this study, longitudinal analyses utilised change scores calculated from W1 to W4. This approach was selected to provide a transparent and interpretable estimate of cognitive change over the cognitive follow-up period and to allow comparison across cognitive domains. Baseline cognitive performance was included as a covariate in adjusted models to account for initial differences in cognitive status.

#### 2.2.5. Covariates

Demographic information, such as the participants’ age, gender, years of education, and non-English-speaking background (NESB), was collected at baseline. Biennially, self-reported questionnaires captured participants’ medical risk factors such as hypertension, dyslipidaemia, smoking status, and diabetes. Height, weight, and blood pressure measurements of participants were also recorded biennially. At each Wave, cardiovascular risk (CVD) was indexed via the Framingham risk score [23]. Participants underwent apolipoprotein E (APOE) genotyping at baseline using DNA extracted from blood. APOE ε2, ε3, and ε4 alleles were distinguished using two single-nucleotide polymorphisms (rs7412 and rs429358) on the TaqMan assay (Applied Biosystems Inc., Thermo Fisher Scientific, Waltham, MA, USA).

### 2.3. Data Analysis

Analysis was performed using IBM SPSS Statistics version 29 (IBM Corp., Armonk, NY, USA). Baseline demographic differences between participants included versus excluded from our sample were compared using chi-square tests for categorical variables and independent *t*-tests for continuous variables to evaluate potential selection bias. Hierarchical linear regressions examined associations between SCC and NPS sum scores and SCC/NPS group status and change in cognitive domains and global cognition over six years (baseline to Wave 4). As six cognitive domains were analysed, domain-specific *p*-values were adjusted for multiple comparisons using a Bonferroni-corrected significance threshold of 0.008. Global cognition was evaluated with the significance set at *p* < 0.05.

#### Dementia Risk Outcomes

Cox proportional hazards models analysed associations with the risk of incident dementia over 12 years (baseline to Wave 7). Time-to-event was defined as the median number of years between the last assessment wave prior to dementia diagnosis and the first assessment wave with a dementia diagnosis. For example, if a participant did not have a dementia diagnosis at Wave 5 (8-year follow-up) and did at Wave 6 (10-year follow-up), their time to event was coded as 9 years. For all models, predictor variables were entered in Step 1. Step 2 additionally included demographic factors (age, gender, education, NESB status) and medical factors (CVD risk score, presence of diabetes and hypertension, and APOE ε4 carrier status), which allowed us to examine the association between the predictor and outcome variables in Step 1 and then examine the robustness of these associations when covariates were introduced.

## 3. Results

### 3.1. Cohort Characteristics

The sample included 468 participants with a mean age of 77.97 years (SD = 4.45); 55.3% were women, and the mean education was 11.82 years (SD = 3.48) (Table 2). APOE ε4 was present in 21.4% of participants, 37.8% had diabetes, and 84.4% had hypertension. The maximum possible CVD risk score was 16, with a mean score of 4.24 (SD = 3.13), and the mean global cognition z-score at baseline was −0.03 (SD = 1.05). SCCs were common, with 70.1% endorsing at least one item. Most participants reported noticing memory changes (70.1%), while only 24.1% were concerned about those changes, and 26.3% had informant-reported memory changes. Most participants endorsed no NPS clusters (80.3%), 17.1% endorsed one cluster, 2.6% endorsed two clusters, and no participant endorsed all three clusters. Among those with NPS, affective symptoms were most common (16.2%), followed by hyperactivity (5.8%), with psychotic symptoms being the rarest (0.2%).

Of the 468 participants in the final sample, 162 participants (34.6%) were classified as having dementia at some point during the study. Of the participants that were not classified as having dementia, 178 (38.0%) were right-censored prior to study completion, and 128 (27.4%) remained dementia-free by Wave 7.

### 3.2. Cognitive Decline

Neither SCC sum nor NPS sum predicted decline in any cognitive domain or global cognition after Bonferroni correction. A marginal association between SCC sum and language decline did not survive correction (β = −0.07, *p* = 0.035; Supplementary Table S2).

Similar hierarchical linear regression models were used to test if SCC/NPS group status predicted change in global cognition and cognitive domains. Group status predicted cognitive decline in the SCC−/NPS+ group only. Relative to the SCC−/NPS− reference group, the SCC−/NPS+ group showed significantly greater decline in visuospatial ability (β = −0.12, *p* = 0.001), which survived Bonferroni correction. Associations with executive function (β = −0.10, *p* = 0.026) and global cognition (β = −0.08, *p* = 0.012) were observed at the uncorrected threshold only. The SCC+/NPS− and SCC+/NPS+ groups did not differ significantly from the reference group on any outcome (Table 3). Age and APOE ε4 status were consistent predictors of steeper decline across most domains.

### 3.3. Incident Dementia

Cox proportional hazards regressions examined whether SCC/NPS group status and SCC and NPS sum scores predicted incident dementia by Wave 7. Each additional SCC item endorsed was associated with a 23% higher hazard of dementia (HR = 1.23, 95% CI [1.03, 1.45], *p* = 0.020); NPS sum was not a significant predictor (Supplementary Table S3). In the group status models, only the SCC+/NPS+ group showed a significantly higher hazard of dementia relative to the SCC−/NPS− reference group (HR = 1.66, 95% CI [1.02, 2.70], *p* = 0.041). The SCC−/NPS+ and SCC+/NPS− groups did not differ significantly from the reference group. Age, APOE ε4 carrier status, and diabetes were significant covariates across both models (Table 4).

### 3.4. Post Hoc Analysis

A post hoc analysis compared baseline characteristics between included (n = 468) and excluded (n = 569) participants (Supplementary Table S4). The groups did not differ significantly on sex, education, NESB status, CVD risk, diabetes, hypertension, or APOE ε4 carrier status. However, excluded participants were older and had lower baseline global cognition. Differences in SCC and NPS endorsement were also observed, suggesting potential selection bias in the final analytic sample.

## 4. Discussion

This longitudinal study explored whether the presence of SCC, NPS, and their combinations was associated with (i) decline in specific cognitive domains, global cognition, and (ii) incident dementia risk in older adults. Partially consistent with our hypotheses, distinct predictive patterns emerged across cognitive and clinical outcomes. The SCC+/NPS+ group demonstrated an elevated risk of incident dementia, but it was not significantly associated with domain-specific decline. The SCC−/NPS+ group showed significant associations with domain-specific cognitive outcomes, including visuospatial ability and, at the uncorrected threshold, executive function and global cognition. Higher SCC sum scores were associated with increased dementia risk but not domain-specific cognitive changes, while NPS sum scores were not independently associated with either cognitive decline or domain-specific cognitive changes. Our study was novel in that it extended the conceptual framework of Ismail and colleagues, incorporating a longer follow-up period of six and 12 years, and examining decline across specific cognitive domains and global cognition [18].

The present study also evaluated simplified operationalisations of SCC and NPS by examining ordinal sum scores to determine whether these conferred more, less, or similar predictive utility. SCC were assessed using a simple ordinal scale that incorporated both self- and informant- reported information. While this specific approach has not been used in prior studies, it is conceptually supported by validated measures of SCC. For instance, the Subjective Cognitive Decline Questionnaire (SCD-Q) consists of two parallel sets of questions for both the participant and the informant [1,3]. Participant-reported cognitive decline was shown to discriminate those with SCD from the control group, whilst informant-reported SCC was a significant predictor of the participant’s objective cognitive performance [4,24,25]. However, the validity of this questionnaire is limited by the lack of longitudinal data and its comparability with other subjective complaint measurement tools.

The SCC−/NPS+ group showed a greater decline in visuospatial ability, with uncorrected associations also observed for executive function and global cognition. However, this group was small, comprising only 18 participants, and was not associated with significantly increased dementia risk. These findings should therefore be interpreted cautiously and considered hypothesis-generating. While the observed pattern may be consistent with literature linking NPS to cognitive vulnerability, the small sample size and absence of corresponding dementia risk prevent strong conclusions about distinct neurodegenerative profiles. The association with worsening executive function observed in the uncorrected models may reflect the early involvement of frontal-subcortical circuits, which are commonly implicated in NPS such as apathy, disinhibition, or depression [26,27]. These networks are critical for executive functioning and attentional control, and their disruption may lead to functional impairments even in the absence of subjective awareness [28,29].

Furthermore, visuospatial deficits are well-established in MCI and have demonstrated strong discriminative power for MCI detection. Neuroimaging work comparing healthy controls and individuals with amnestic-MCI has shown that the largest difference was present in visuospatial skills [30,31]. Analysis of the visuospatial networks showed early alterations in frontotemporal-visuospatial networks (e.g., reduced cortical thickness in the temporal pole and superior temporal gyrus; [30,32]. Our finding that the NPS-only group exhibited a decline in visuospatial ability even prior to MCI suggests that these structural and functional changes may emerge even earlier in the disease trajectory.

Notably, having NPS only was not associated with increased risk of incident dementia, despite showing the steepest decline across several individual cognitive domains. This apparent dissociation suggests that short- to medium-term domain-specific cognitive decline and longer-term clinical conversion may capture different aspects of cognitive ageing and disease progression. Elevated NPS in the absence of SCC may identify individuals with measurable cognitive vulnerability, but not necessarily a trajectory that translates into dementia diagnosis within the follow-up period. The predominance of executive and visuospatial decline, rather than a primarily memory-led pattern, may also point to a more heterogeneous cognitive profile that is less closely aligned with typical pathways to incident dementia. Although NPS may reflect early neurobiological or psychosocial vulnerability in some individuals, the present findings do not allow us to determine whether the SCC−/NPS+ group represents a distinct neurodegenerative phenotype. Replication in larger samples with biomarker data and more detailed NPS measures is required.

Aligning with our hypothesis, participants with both SCC and NPS (SCC+/NPS+) had the greatest increase in dementia risk. This corresponds with Ismail et al. [18], despite important differences in how NPS were defined. Specifically, Ismail et al. used the Mild Behavioural Impairment (MBI) construct, which requires symptoms to persist for at least six months [18,33]. In contrast, our operationalisation was intentionally less restrictive, requiring only the presence of at least one SCC and one NPS at baseline, without evidence of symptom persistence over time. The fact that this simpler cross-sectional classification was still associated with increased dementia risk suggests that routinely collected clinical and cohort measures, such as brief SCC items and the NPI, may provide meaningful information about future risk when considered together.

The null findings for the SCC-only (NPS−/SCC+) group may reflect the heterogeneity of subjective cognitive complaints. In some individuals, SCCs may reflect non-neurodegenerative influences such as mood fluctuations, physical health concerns, or heightened self-monitoring. In others, SCCs may represent an earlier point in the disease process, before objective cognitive decline or clinical conversion is detectable [34,35]. This distinction is important because it suggests that SCCs may be most prognostically informative when interpreted alongside other behavioural or clinical indicators, rather than in isolation.

We also examined whether SCC and NPS composite scores provided additional insight into cognitive decline and dementia risk. SCC sum score showed a modest association with language decline, although this did not survive correction for multiple comparisons. This pattern is broadly consistent with studies linking SCC to language deterioration, including evidence that subjective cognitive changes may be experienced as word-finding difficulties [36,37,38]. However, the absence of a corrected association suggests that this finding should be interpreted cautiously.

In contrast to previous studies linking individual neuropsychiatric symptoms to specific cognitive deficits, such as anxiety with executive dysfunction and apathy with memory decline, the NPS sum score was not significantly associated with decline in any individual cognitive domain [39,40]. This discrepancy may reflect the way the NPS burden was operationalised in the present study. We used a cluster-based summary approach rather than modelling individual symptom frequency and severity. While this approach provides a pragmatic measure of overall NPS burden and is well-suited to cohort data, it may lack the specificity needed to detect symptom-specific cognitive profiles. Future studies using more granular NPS measures may be better placed to clarify whether particular symptoms, or symptom clusters, are differentially associated with decline across cognitive domains.

In the Cox regression model, the SCC sum score was a significant independent predictor of incident dementia. Each additional SCC item endorsed was associated with approximately 23% higher dementia risk, suggesting that the cumulative burden of subjective complaints carries prognostic value beyond the presence or absence of complaints alone. This finding is consistent with the SCD-plus framework, which proposes that particular features of subjective cognitive decline are more strongly associated with future clinical progression than non-specific complaints. For example, concern about cognitive decline has been linked to increased progression risk [1,4,41]. Jessen et al. [4] reported that SCC accompanied by concern was associated with a 3.5-fold greater three-year risk of progression, while SCC without concern was associated with an almost two-fold increased risk compared to non-complainers. Informant confirmation of cognitive decline also appears to provide important prognostic information and is often a stronger predictor of future impairment than self-report alone [42,43]. In line with this, Numbers et al. [43] showed that while participants’ baseline level of SCC reporting was associated with dementia risk, both informants’ baseline reporting and increase in reporting SCC over time were significant predictors of incident dementia.

In contrast, NPS sum scores were not significantly associated with dementia risk in the present model. This differs from previous literature showing that specific neuropsychiatric symptoms, including agitation, apathy, anxiety, and irritability, are associated with increased dementia risk across a range of samples [44,45,46]. However, this discrepancy may reflect the operationalisation of NPS in the present study. The NPS sum score captured the number of symptom clusters endorsed at baseline, but did not account for symptom type, frequency, severity, or persistence. This broad measure may therefore lack the specificity needed to identify the particular neuropsychiatric profiles most relevant to dementia risk. The NPS sum score should also be interpreted cautiously, given its limited variability in this cohort. Most participants endorsed no NPS clusters, relatively few endorsed more than one cluster, and no participants endorsed all three clusters. As such, the NPS sum score may not have provided sufficient spread to adequately test dose-response relationships between NPS burden and cognitive or dementia outcomes. The absence of significant associations for NPS sum score may therefore reflect limited variability and low symptom prevalence, rather than evidence that NPS burden is unrelated to later cognitive outcomes. In contrast, SCC sum scores may more directly reflect cumulative perceived cognitive change, making it a more sensitive predictor of clinical progression in this cohort.

A key strength of this study is the use of a large, well-characterised, community-based cohort, which reduces the likelihood of clinical referral bias and strengthens the reliability of the findings. Cognition was assessed comprehensively using detailed neuropsychological, psychiatric, and clinical data, enabling examination of both global and domain-specific cognitive change. The inclusion of both self- and informant-reported measures also allowed us to capture multiple dimensions of cognitive and behavioural change. In particular, the informant-rated NPI enabled identification of neuropsychiatric symptoms that participants may not have recognised or reported themselves. The use of established NPS clusters, supported by previous clinical and factor-analytic work, provided a clinically meaningful framework for characterising behavioural symptoms. Another major strength is the 12-year follow-up period, which reduced the likelihood of spurious short-term associations and allowed examination of longer-term dementia risk. By directly comparing subjective and behavioural indicators within the same cohort, this study provides a more nuanced understanding of early dementia risk phenotypes than studies examining either construct in isolation. To our knowledge, this is the first study to evaluate the joint and independent associations of SCC and NPS with both domain-specific cognitive decline and incident dementia, highlighting their complementary but distinct prognostic value.

Several limitations should be considered when interpreting these findings. First, MAS participants were recruited from two electorates in Sydney and were predominantly Caucasian, well-educated, and relatively socioeconomically advantaged community-dwelling older adults. This limits generalisability to more diverse and representative older Australian populations. Attrition and selection bias should also be considered. The final analytic sample represented less than half of the original MAS cohort, and participants excluded from the present analyses differed from those included on several baseline characteristics. In particular, excluded participants were older and had lower baseline global cognition, suggesting that the final sample may represent a healthier and more cognitively intact subgroup. Differences in SCC and NPS endorsement between included and excluded participants also indicate that the analytic sample may not fully capture the range of subjective and neuropsychiatric symptoms present in the original cohort. This selective retention may have attenuated associations between SCC, NPS, cognitive decline, and incident dementia, and the reported effects may therefore be conservative estimates.

Cognitive decline was operationalised using change scores from baseline to Wave 4. While this approach provides a transparent and interpretable measure of six-year change, change-score analyses can be influenced by measurement error and regression to the mean. Future studies could use longitudinal mixed-effects modelling or other repeated-measures approaches to better account for individual variability in cognitive trajectories over time. SCC sum scores were analysed under the presumption that each item had equal weight. However, some items (e.g., concern, informant report) may carry greater prognostic value, which could not be explored here. SCC items were also binary, potentially underestimating variability in severity or frequency. Finally, both SCC and NPS were assessed cross-sectionally at baseline, limiting interpretation of temporal dynamics or persistence of symptoms. The simplified operationalisation of SCC and NPS is both a strength and a limitation. Brief binary SCC items and NPS symptom clusters reflect the type of information commonly available in large cohort studies and may support pragmatic risk characterisation. However, this approach does not capture symptom severity, frequency, persistence, or symptom-specific effects, and should not be considered equivalent to formal SCD or MBI diagnostic criteria. Future studies using more detailed and repeated SCC and NPS measures may clarify whether more granular symptom profiles improve the prediction of cognitive decline and dementia.

Although these findings suggest that SCC and NPS may provide complementary information about dementia risk, predictive performance was not directly evaluated. We did not assess discrimination, sensitivity, specificity, or positive and negative predictive values. Therefore, the present findings should be interpreted as supporting further investigation of SCC and NPS for risk stratification, rather than as evidence for immediate implementation in clinical screening protocols. Although some statistically significant associations were observed, several effect sizes were modest. The clinical meaningfulness of these effects, therefore, remains uncertain, particularly for individual-level prediction. Future studies should evaluate whether SCC and NPS measures improve prediction beyond established demographic, genetic, and clinical risk factors, and whether these associations translate into clinically useful risk stratification. Expanding analyses across diverse cohorts, with biomarker integration, would also strengthen the generalisability and mechanistic understanding. Intervention trials targeting individuals with isolated NPS or SCC-plus features may help determine whether these represent modifiable early stages of neurodegenerative disease.

## 5. Conclusions

These findings suggest that SCC and NPS provide complementary but distinct information regarding cognitive and clinical trajectories in older adults. SCC burden was associated with increased dementia risk, while NPS in the absence of SCC was associated with greater visuospatial decline, although this latter finding should be interpreted cautiously, given the small subgroup size. The co-occurrence of SCC and NPS identified a group with elevated dementia risk, supporting further investigation of multidimensional risk profiles in preclinical dementia research. However, predictive performance metrics were not evaluated, and prospective validation studies are needed before these measures can be recommended for clinical screening or decision-making.

## Figures and Tables

**Table 1:** Grouping of NPS symptoms into clusters. Adapted from Sayegh and Knight [22].

NPS Cluster	Neuropsychiatric Symptoms
Psychotic (NPS-Psy)	Delusions Hallucinations
Affective (NPS-Aff)	Depression Anxiety Apathy Sleep disturbance Appetite disturbance
Hyperactivity (NPS-Hyp)	Agitation Irritability Disinhibition

SCC and NPS sum scores were intended to provide pragmatic indicators of baseline symptom burden. These scores should therefore be interpreted as simplified measures of complaint and symptom endorsement, rather than as measures of symptom severity, frequency, persistence, or diagnostic SCD or MBI status.

**Table 2:** Baseline participant demographics, predictors, and outcome variables (n = 468).

Variables	n (%) or M ± SD
**Demographics**	
Age (years)	77.97 ± 4.45
Gender, women	259 (55.3%)
Education (years)	11.82 ± 3.48
NESB status	83 (17.7%)
CVD risk score ^a^	4.24 ± 3.13
Diabetes ^b^	177 (37.8%)
Hypertension ^b^	395 (84.4%)
APOE ε4 carrier status	100 (21.4%)
**Subjective Cognitive Complaints (SCC)**	
SCC-item 1 SCC-item 2 SCC-item 3	328 (70.1%) 113 (24.1%) 123 (26.3%)
SCC sum score (n, %) 0 1 2 3	140 (29.9%) 146 (31.2%) 128 (27.4%) 54 (11.5%)
**Neuropsychiatric Symptoms (NPS)**	
Endorsed any NPS (yes) NPS-Psy NPS-Aff NPS-Hyp	92 (19.7%) 1 (0.2%) 76 (16.2%) 27 (5.8%)
NPS sum score (n %) 0 1 2 3	376 (80.3%) 80 (17.1%) 12 (2.6%) 0 (0.0%)
**SCC/NPS Group Status (n, %)**	
SCC−/NPS−	122 (26.1%)
SCC+/NPS−	254 (54.3%)
SCC−/NPS+	18 (3.8%)
SCC+/NPS+	74 (15.8%)
**Cognitive Domains ^†^ (M ± SD)**	
Attention/Processing Speed	0.04 ± 0.99
Language	−0.13 ± 1.10
Executive function	0.07 ± 1.00
Visuospatial	0.00 ± 1.01
Memory	−0.02 ± 1.04
Global cognition score	−0.03 ± 1.05

Note: NESB = Non-English-Speaking Background; CVD = cardiovascular disease; APOE ε4 = apolipoprotein ε4 status; SCC = Subjective Cognitive Complaints; NPS = Neuropsychiatric Symptoms; NPS-Psy = Psychotic Neuropsychiatric Symptoms Cluster; NPS-Aff = Affective Neuropsychiatric Symptoms Cluster; NPS-Hyp = Hyperactivity Neuropsychiatric Symptoms Cluster. ^a^ Not adjusted for age. ^b^ Currently present or ever had. ^†^ Quasi z-scores based on baseline MAS norms.

**Table 3:** Fully adjusted hierarchical regression model examining associations between group status and six-year change in cognitive domains and global cognition scores.

Cognitive Domains	Fully Adjusted Models ^a^			
	B	95% CI (LL, UL)	β	*p*
**Global Cognition**				
SCC−/NPS− (reference group)	-	-	-	-
SCC+/NPS−	−0.13	[−0.32, 0.06]	−0.05	0.187
SCC−/NPS+	−0.53	[−0.94, −0.12]	−0.08	0.012
SCC+/NPS+	−0.20	[−0.46, 0.05]	−0.06	0.117
**Attention/Processing Speed**				
SCC+/NPS−	−0.14	[−0.37, 0.09]	−0.06	0.220
SCC−/NPS+	−0.50	[−1.03, 0.03]	−0.08	0.062
SCC+/NPS+	−0.23	[−0.53, 0.08]	−0.07	0.143
**Language**				
SCC+/NPS−	0.01	[−0.18, 0.19]	<0.01	0.956
SCC−/NPS+	−0.04	[−0.46, 0.38]	>−0.01	0.849
SCC+/NPS+	−0.17	[−0.42, 0.09]	−0.05	0.192
**Executive Function**				
SCC+/NPS−	−0.23	[−0.53, 0.07]	−0.07	0.137
SCC−/NPS+	−0.77	[−1.44, −0.09]	−0.10	0.026
SCC+/NPS+	−0.16	[−0.56, 0.25]	−0.04	0.445
**Memory**				
SCC+/NPS−	−0.15	[−0.35, 0.04]	−0.06	0.115
SCC−/NPS+	−0.23	[−0.64, 0.19]	−0.04	0.280
SCC+/NPS+	−0.23	[−0.49, 0.02]	−0.07	0.074
**Visuospatial**				
SCC+/NPS−	0.02	[−0.16, 0.22]	0.01	0.799
SCC−/NPS+	−0.71	[−1.14, −0.29]	−0.12	0.001
SCC+/NPS+	−0.06	[−0.31, 0.20]	−0.02	0.665

Note: SCC = subjective cognitive complaints; NPS = neuropsychiatric symptoms; CI = confidence interval; LL = lower limit; UL = upper limit; ^a^ Controlling for participant, age, sex, education, NESB status, Wave 1 cognition scores, diabetes, hypertension, APOE ε4 status, CVD risk.

**Table 4:** Hierarchical Cox proportional hazards models examining relationships between group status, demographic and clinical risk factors, and risk of incident dementia over 12 years.

Variables			
	HR	95% CI (LL, UL)	*p*
**SCC**−**/NPS−** (reference group)	-	-	0.168
**SCC+/NPS−**	1.14	[0.76, 1.71]	0.517
**SCC−/NPS+**	1.57	[0.69, 3.59]	0.281
**SCC+/NPS+**	1.66	[1.02, 2.70]	0.041
**Gender**	0.80	[0.58, 1.12]	0.203
**Age**	1.12	[1.08, 1.16]	<0.001
**Education**	1.01	[0.96, 1.06]	0.666
**NESB status**	1.20	[0.81, 1.77]	0.368
^a^ **CVD risk**	0.99	[0.94, 1.05]	0.757
^b^ **Diabetes**	1.45	[1.03, 2.04]	0.034
**Hypertension**	1.07	[0.61, 1.88]	0.818
**APOE ε4 carrier**	2.08	[1.46, 2.95]	<0.001

Note: SCC = Subjective Cognitive Complaints; NPS = Neuropsychiatric Symptoms; NESB = Non-English-Speaking Background; CVD = Cardiovascular Disease; APOE ε4 = Apolipoprotein ε4 Status; HR = Hazard Ratio; CI = Confidence Interval; LL = Lower Limit; UL = Upper Limit; ^a^ Not adjusted for age. ^b^ Currently present or ever had.

## Data Availability

The terms of consent for research participation stipulate that an individual’s data can only be shared outside of the Memory and Ageing Study investigators’ group if the group has reviewed and approved the proposed secondary use of the data. This consent applies regardless of whether data have been de-identified. Access is mediated via a standardised request process managed by the CHeBA Research Bank, which can be contacted at ChebaData@unsw.edu.au.

## Supplementary Materials

The following supporting information can be downloaded at: https://www.mdpi.com/article/10.3390/brainsci16070693/s1, Table S1: Corresponding neuropsychological tests for each cognitive domain; Table S2: Associations between SCC and NPS sum scores and six-year change in cognitive domains and global cognition; Table S3: Association between SCC and NPS sum scores, demographic and clinical risk factors, and incident dementia risk over 12 years; Table S4: Baseline characteristics of included (n = 468) versus excluded (n = 569) participants.
