# Supplementary material for: Combined Influence of Subjective Cognitive Complaints and Neuropsychiatric Symptoms on Cognitive Trajectories in Older Adults
Source: Brain Sci. 2026 Jun 30;16(7):693. doi: 10.3390/brainsci16070693 (PMC13406594; doi:10.3390/brainsci16070693)
Supplement: Supplementary file 1 [file brainsci-16-00693-s001.zip › brainsci-4360580-supplementary.pdf]

Supplementary Materials

Supplementary Table S1.
Corresponding neuropsychological tests for each cognitive domain.

Cognitive Domain	Neuropsychological Test
Attention Processing Speed	Digit Symbol-Coding<br>Trail Making Test A
Language	Boston Naming Test<br>Semantic Fluency (Animals)
Executive Function	Controlled Oral Word Association Test<br>Trail Making Test B
Memory	Logical Memory Story A delayed recall<br>Rey Auditory Verbal Learning Test<br>Benton Visual Retention Test recognition
Visuospatial	Block Design

Supplementary Table S2.
Associations between SCC and NPS sum scores and six-year change in cognitive domains and global cognition.

Domain	Predictor	B	95% CI	*β*	*p*
†Global Cognition	SCC sum	-0.07	[-0.15, 0.02]	-0.05	.124
	NPS sum	-0.10	[-0.27, 0.08]	-0.03	.280
Attention/Processing Speed	SCC sum	-0.06	[-0.17, 0.04]	-0.05	.209
	NPS sum	-0.12	[-0.33, 0.10]	-0.04	.277
Language	SCC sum	-0.09	[-0.17, -0.01]	-0.07	.035*
	NPS sum	-0.06	[-0.23, 0.12]	-0.02	.511
Executive Function	SCC sum	-0.05	[-0.18, 0.08]	-0.03	.457
	NPS sum	-0.06	[-0.33, 0.22]	-0.02	.697
Memory	SCC sum	-0.07	[-0.15, 0.01]	-0.06	.091
	NPS sum	-0.06	[-0.24, 0.11]	-0.02	.483
Visuospatial	SCC sum	0.01	[-0.07, 0.10]	0.01	.787
	NPS sum	-0.13	[-0.31, 0.04]	-0.06	.135

Note: Fully adjusted for age, gender, education, NESB status, Wave 1 domain score, diabetes, hypertension, APOE ε4 status, CVD risk. *p<.05 significant at uncorrected threshold. †Quasi z-scores based on baseline MAS norms.

Supplementary Table S3.
Association between SCC and NPS sum scores, demographic and clinical risk factors, and incident dementia risk over 12 years.

	HR	95% CI	p
SCC sum	1.23	[1.03, 1.45]	.020*
NPS sum	1.32	[0.97, 1.79]	.078
Gender	0.85	[0.61, 1.19]	.345
Age	1.12	[1.08, 1.16]	<.001**
Education	1.01	[0.97, 1.06]	.597
NESB status	1.21	[0.82, 1.77]	.346
[a]CVD risk	0.99	[0.94, 1.05]	.687
Diabetes	1.49	[1.06, 2.09]	.023*
Hypertension	1.06	[0.60, 1.87]	.829
APOE ε4 carrier	1.94	[1.35, 2.77]	<.001**

Note: SCC=Subjective Cognitive Complaints; NPS=Neuropsychiatric Symptoms; NESB=Non-English-Speaking Background; CVD=cardiovascular disease; APOE=apolipoprotein E; HR=hazard ratio; CI=confidence interval. [a]Not adjusted for age. *p<.05 significant at uncorrected threshold; **p<.001 significant at corrected threshold.

Supplementary Table S4
Baseline characteristics of included (n = 468) versus excluded (n = 569) participants.

Variable	Included n = 468	Excluded n = 569	p
Age, years	77.92 ± 4.45	78.62 ± 5.07	.029*
Gender, women	259 (55.3%)	313 (55.0%)	.915
Education, years	11.82 ± 3.48	11.42 ± 3.46	.063
NESB status	83 (17.7%)	81 (14.2%)	.124
[a]CVD risk score	4.24 ± 3.13	4.18 ± 3.13	.377
[a]Diabetes	177 (37.8%)	225 (39.5%)	.809
Hypertension	395 (84.4%)	492 (87.0%)	.385
APOE ε4 carrier	100 (21.6%)	122 (23.4%)	.518
[†]Global cognition	-0.03 ± 1.05	-0.29 ± 1.13	<.001**
Endorsed any SCC	328 (70.1%)	289 (59.8%)	<.001**
Endorsed any NPS	92 (19.7%)	164 (32.5%)	<.001**

Note: NESB = Non-English-Speaking Background; CVD = cardiovascular disease; APOE = apolipoprotein E; SCC = Subjective Cognitive Complaints; NPS = Neuropsychiatric Symptoms. [a]Not adjusted for age. [b] Current presence or ever had. [†]Quasi z-scores based on baseline MAS norms. *p<.05 significant at uncorrected threshold; **p<.001 significant at corrected threshold.
